# Impact of Previous Irradiation on Wound Healing after Negative Pressure Wound Therapy in Head and Neck Cancer Patients—A Systematic Review

**DOI:** 10.3390/cancers13102482

**Published:** 2021-05-19

**Authors:** Muhammad Faisal, Peter D. Berend, Rudolf Seemann, Stefan Janik, Stefan Grasl, Andrea Ritzengruber, Herbert Mendel, Arif Jamshed, Raza Hussain, Boban M. Erovic

**Affiliations:** 1 Institute of Head and Neck Diseases, Evangelical Hospital, 1180 Vienna, Austria; maxfas@live.com (M.F.); rudolf.seemann@gmail.com (R.S.); 2 Department of Head and Neck Surgery, Shaukat Khanum Memorial Cancer Hospital, Lahore 54000, Pakistan; jamshedarif@gmail.com (A.J.); razah@skm.org.pk (R.H.); 3 Department of Otolaryngology, Head and Neck Surgery, Medical University of Vienna, 1090 Vienna, Austria; pdberend@gmail.com (P.D.B.); stefan.janik@meduniwien.ac.at (S.J.); stefan.grasl@meduniwien.ac.at (S.G.); 4 Wound Management, Evangelical Hospital, 1180 Vienna, Austria; a.ritzengruber@gmail.com; 5 Department of General Surgery, Evangelical Hospital, 1180 Vienna, Austria; herbert.mendel@gmx.at

**Keywords:** negative pressure wound therapy, head and neck cancers, radiation therapy

## Abstract

**Simple Summary:**

The study has focused on the use of Negative Pressure Wound Therapy (NPWT) specifically in head and neck cancer patients. The sole purpose of using NPWT is to expedite the process of healing as most of the head and neck cancer patients are bound to get post-operative radiotherapy and any delay would further lengthen the treatment and affect the outcome. Previous irradiation and Diabetes Mellitus have detrimental impact on wound healing after NPWT.

**Abstract:**

(1) Background: Negative pressure wound therapy (NPWT) has been effectively used for wound management in comparison to traditional dressings. The purpose of this study was to provide an evidence-based review of NPWT in head and neck cancer patients, as well as the impact of previous irradiation and other risk factors on wound healing. (2) Material and Methods: We conducted a comprehensive search in PubMed, Medline, Embase, Web of Science, and Cochrane Library databases for relevant literature. (3) Results: 15 studies fulfilled the inclusion criteria. The most common etiologies requiring NPWT were defects post tumor resection and flap reconstruction and oro/pharyngo-cutaneous fistulas. The neck was found to be the most common site of involvement (47.3%). The overall wound healing response rate was 87.5%. The median negative pressure recorded was 125 mm of Hg, with a median dressing change time of three days. Previous irradiation (*p* = 0.01; OR = 4.07) and diabetes mellitus (DM) (*p* = 0.001; OR = 5.62) were found to be significantly associated with delayed wound healing after NPWT. (4) Conclusion: NPWT treats complex wounds in head and neck cancer patients and should represent a significant armamentarium in head and neck cancers. Previous irradiation and DM have detrimental effects on wound healing after NPWT.

## 1. Introduction

Negative pressure wound therapy (NPWT) has remained an integral part of wound management for general, orthopedic, and plastic surgeons for more than half a decade [1,2,3,4]. Adjustable negative pressure applied via an adhesive film over a foam padding promotes wound healing by removing wound exudate and decreasing interstitial edema and bacterial load at the wound site. This results in increased tissue perfusion and promotes the formation of a well-granulating wound bed [5,6,7]. Though NPWT has been used in the management of donor site morbidity in head and neck reconstruction, limited data on its use in head and neck sites have been published. This may be attributed to the complexity of anatomical subsites of the head, the neck, and (subsequently) the difficulty of properly adjusting a NPWT dressing. The majority of head and neck cancer patients present at an advanced stage, thus requiring major ablative surgeries and the reconstruction of large defects. Poor functional status, malnutrition, previously irradiated tissue, and poor oral hygiene contribute to compromised wound healing that requires additional support for wound management [8]. Delayed wound healing has its own share of financial burden- and treatment-related delays that significantly impact clinical outcomes. The purpose of this comprehensive review was to give a detailed analysis of the use of NPWT in head and neck cancer patients, as well as to analyze the impact of previous irradiation and other risk factors on patients’ wound healing.

## 2. Results

### 2.1. Search Findings

**Figure 1:**
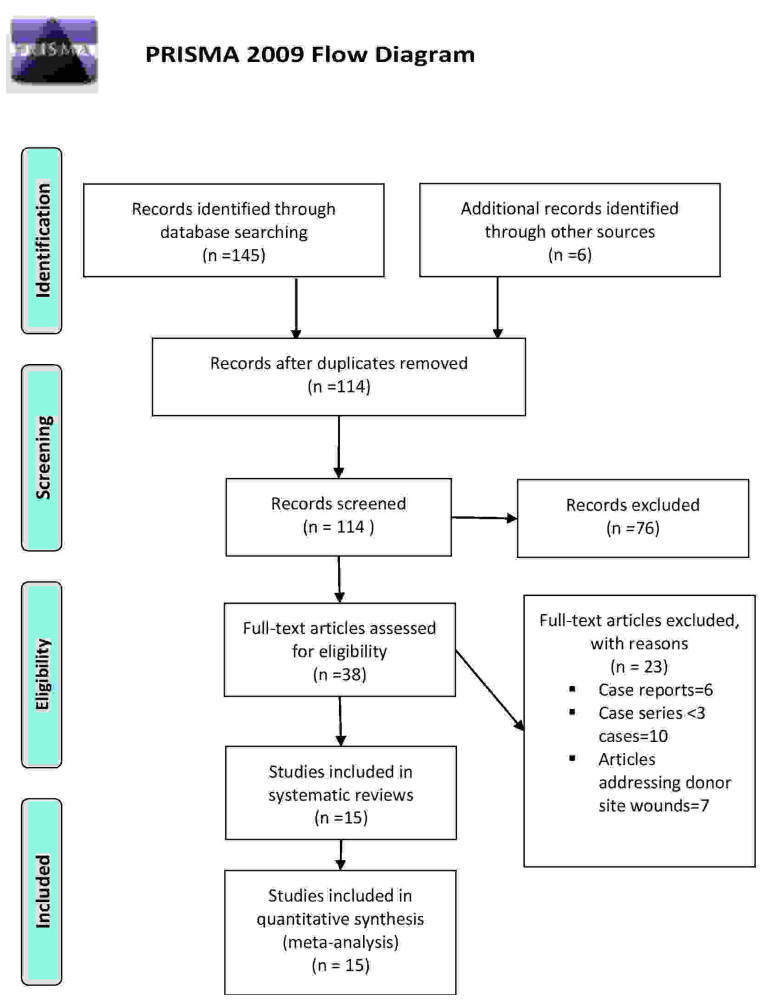
The flow diagram was adapted from PRISMA recommendations.

A total of 151 articles were identified with the database search by using keywords, as mentioned above. One hundred and fourteen (114) articles remained after duplication removal. The articles were further screened by title and abstract reading to select the relevant studies. Short case series reporting less than 3 patients were further excluded. After full-text revision, 23 articles were excluded for various reasons explained in the PRISMA flow chart [8,9,10,11,12,13,14,15,16,17,18,19,20,21,22]. Hence, 15 studies published in English and 1 published in German were included for further investigation. We followed the PRISMA guidelines, and the study selection procedure is illustrated by the PRISMA flow diagram shown in Figure 1.

### 2.2. Study Cohort

There were a total of 380 patients with a mean age of presentation of 63 years (range: 50–67), with cumulative results comprising 287 males and 93 females at a ratio of 3:1 (Table 1). The therapy has found the majority of its applications for wounds in the neck region (n = 170; 47.3%), followed by the face (n = 66; 18.3%) and the head (n = 20; 5.5%).

### 2.3. NPWT Therapy

**Figure 2:**
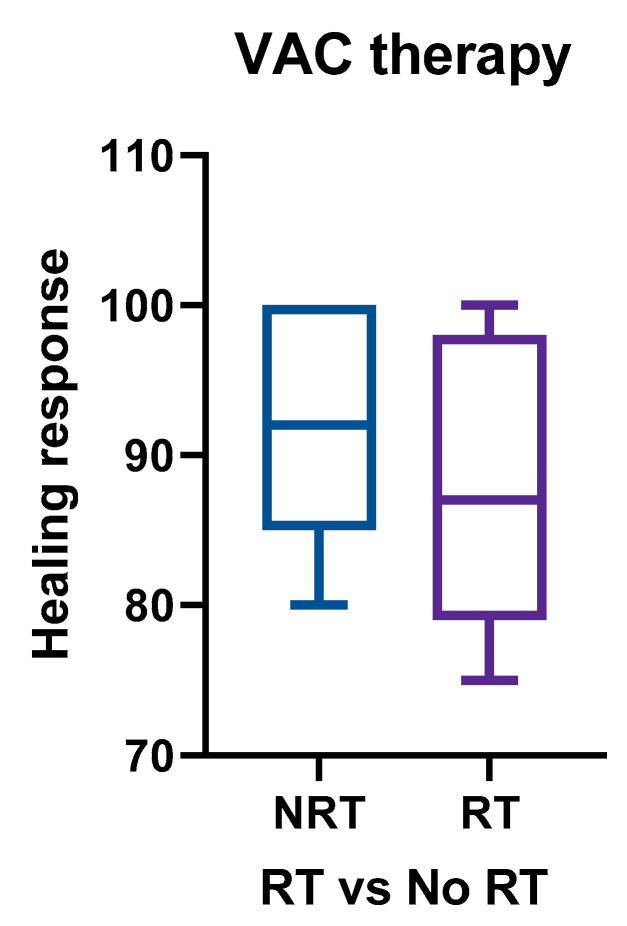
The mean healing response using NPWT therapy in the absence of previous irradiation was found to be 92.40 ± 8.29, compared to 86.7 ± 11.7 with previous irradiation. Abbreviation: RT: radiotherapy; NRT: no radiotherapy; NPWT: negative pressure wound therapy.

The mean wound healing response was 88.2% (82.2 ± 10.4%). The intended goal of NPWT was either granulation tissue formation (thus providing a bed for skin grafting) or complete wound closure, while the wounds that failed to meet this standard benchmark required additional small procedures to carry out wound closure. Approximately half of the studies in our cohort reported healing time with a varied range (Table 1). The mean times for healing and dressing change was 14.4 days (range: 6–53) and 3 days (range: 2–5), respectively. Among the reported factors or comorbidities that are thought to be responsible for delayed wound healing are previous irradiation (39.8%), diabetes mellitus (DM) (22%), hypertension (HTN) (19.4%), hypothyroidism (11.97%), malnutrition (4.17%), and coronary artery disease (CAD) (3.8%) (Table 1). The mean duration of hospital stay in our cohort was 10 days, with a wide range (2–35 days) because the patients with high output chyle or associated multiple comorbidities stayed longer. Only 4 studies reported a treatment cost that ranged between 100 and 250 USD. The use of regional or free flaps in our cumulative series did not seem to impact the outcome in terms of wound healing. The etiology of dehiscence was attributed to the compromised blood supply at the recipient site post irradiation. The diminished recipient site vascularity in combination with poor suturing technique and immunosuppressed status was found to usually predispose the wounds to early breakdown. The mean healing response observed in group A (comprising studies where patients received no radiotherapy) was 92.40 ± 8.29%, which that of group B (comprising patients where RT was used) was recorded at 86.7 ± 11.7% (Figure 2).

### 2.4. Risk Factors for Delayed Wound Healing

Univariate and covariate binary logistic regression analyses were performed to analyze the impact of risk factors on wound healing. The univariate analysis showed the significant detrimental effect of previous irradiation on wound healing (*p* = 0.005; OR = 4.34). Similarly, the presence of comorbid conditions such as DM also resulted in a significantly decreased wound healing response (*p* = 0.01; OR = 5.65). Other comorbidities such as malnutrition, peripheral vascular disease (PVD), and hypothyroidism have not shown any significant impact on wound healing in NPWT patients. Furthermore, multivariate analyses have shown previous radiation and DM to be the only significant factors that alter wound healing. (Table 2).

### 2.5. Quality of Studies

The quality of assessment for the selected retrospective studies and case series was performed using National Institutes of Health Quality Assessment Tools. The quality was calibrated from 9 parameters: study design, study objectives, sample size justification, level of exposure to outcome, clearly defined outcomes, statistical justification, follow up, and confounding variables. Twelve studies qualified as good, while three were fair in assessment criteria (Table 3).

## 3. Discussion

Wound management protocols have evolved over centuries, starting from the application of hot beer and water and moving to bandages using plaster and herbs; mixtures of honey, paste, and lint; debridement and amputations in war of affected zones; and the advent of antibiotics as knowledge of immunology and microbiology was acquired in the 20th century [24]. The concept of using sub-atmospheric pressure was coined by Fleischman in 1993 [25]. Initially, the idea was utilized in the treatment of open fractures and acute, chronic, and fasciotomy wounds [26,27,28].

The published literature has focused on the use of dressings for sacral, sternal, upper and lower extremity, perineal, and abdominal wounds. Even studies related to head and neck cancer wounds have been more targeted to donor site management after free flap reconstruction [29]. Though the literature is limited in the form of case reports, case series, and retrospective single arm cohorts, it has still provided us with a useful information in terms of dressings utilization, constraints, indications, contraindications, and factors affecting outcome. We tried to focus our discussion on the management of head and neck cancer-related primary site wounds because their anatomical complexities have posed challenges to treating physicians.

The presence of co-morbidities, alcohol, smoking, and malnutrition as risk factors have already been established as contributing to compromised wound healing in head and neck cancer patients. The risk is further aggravated by the presence of the bacterial flora of the upper aerodigestive tract. This not only increases the financial burden on the health care system but also has resulted in adjuvant treatment delays that impact survival outcomes [30]. Previous irradiation, DM, HTN, and hypothyroidism were the most common factors shared among our cohort, with only five studies reporting no previous irradiation and 10 studies documenting one or more than one comorbidity in the majority of patients in the background of previous irradiation.

**Figure 3:**
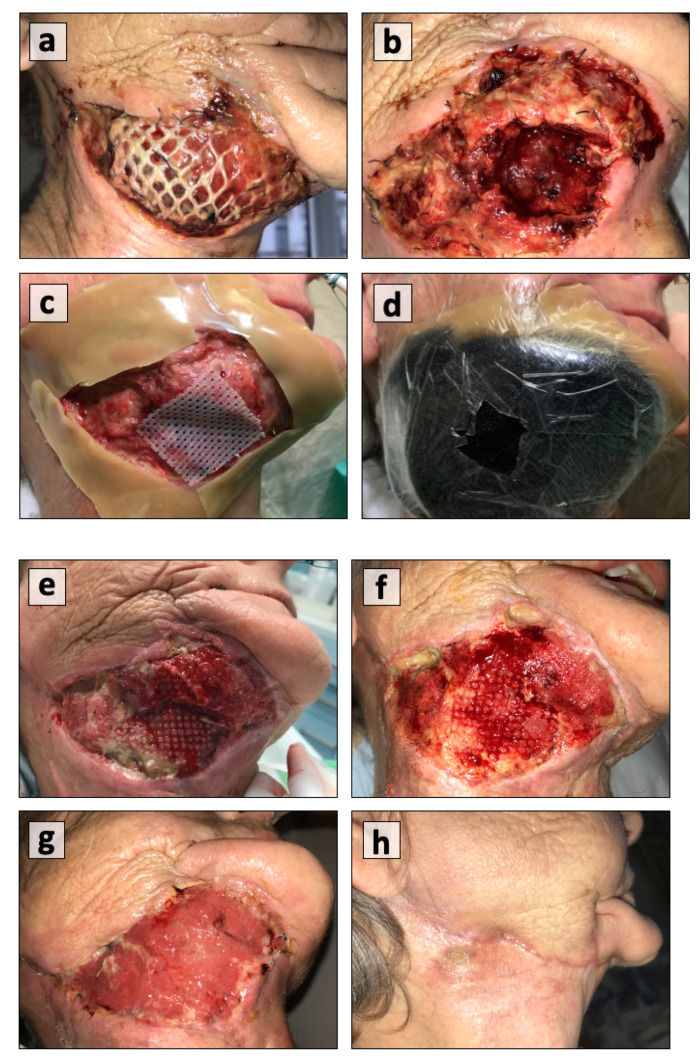
(**a**) Pectoralis major muscle flap coverage for reconstruction plate that developed hematoma and infection; (**b**) evacuation of hematoma and debridement; (**c**) the placement of a hydrocolloid film around the wound margins and a grid to protect the thoracodorsal vessels of the flap; (**d**) the placement of a polyurethane foam dressing with an open pore structure; (**e**) good blood flow and granulation tissue formation after 1st dressing; (**f**) uniform healing at 2nd dressing; (**g**) wound healing at 3rd week; and (**h**) wound healing after 4 months.

When applied to extremities, these dressings usually have a large surface area and no anatomical restrictions. They serve the purpose of granulation tissue formation for potential skin grafting and infection control. In contrast, face or oral cavity wounds usually have large dead spaces and through-and-through defects. Large defect sizes, the dynamics of the recipient site, the poor viability of adjacent tissue due to previous irradiation, and faulty suturing techniques usually contribute to orofacial or orocervical communication. Yang et al. recommended the use of a water-tight seal at the mucosal side and the application of NPWT dressing on the skin side for air sealing and continuous function [16]. They also advocated for delaying NPWT application for two weeks around the free flap to avoid any compromises in flap perfusion by the pedicle. Lin et al. found it safe to immediately apply NPWT after flap reconstruction. Another frequent indication for the use of NPWT in the head and neck are pharyngocutaneous fistulas post laryngectomy or pharyngolaryngectomy [8]. Different approaches have been used for the management of fistula tracts in the pharynx with the use of either conventional external foam dressing attached to a vacuum device or the use of intraluminal NPWT for the closure of the tract or the optimization of pharyngeal reconstruction [11,15]. Conventional daily dressing management is a cumbersome process involving a prolonged hospital stay and an additional financial burden on the healthcare system, with no convincing results. The undulating wound surfaces and movements of the neck pose problems of air leak and frequent dressing change with no effective outcome. Small maneuvers, such as the shallow insertion of foam dressing in the fistulous tract, the use of a tracheostomy tube with bulb inflation, a slight increase in negative pressure for large sized openings, bending or slightly turning the neck to the ipsilateral side, and the use of intraluminal foam dressings if the fistula is too large, seem to be successful [15,31]. Similarly, there is controversy regarding the placement of NPWT instead of synthetic or inert materials such as reconstruction titanium plates, synthetic vascular grafts, and mesh. Dhir et al. have documented successful use of NPWT in these scenarios, but there have been case reports and series of failed wound healing over titanium plates [13,32,33]. In this paper, we have reported a case of plate coverage at the mandible–neck junction using the pectoralis major (PM) muscle flap with overlying skin grafting (Figure 3). The development of hematoma and infection resulted in the failure of the external flap surface, including the graft. The wound was debrided and NPWT was applied using a continuous pressure of 125 mm of Hg. The defect completely healed, with the filling of the dead space occurring over a period of three weeks. In contrast, the placement of vacuum dressing in another patient with plate exposure at the para-symphysis area failed to give the desired results. Multiple factors, such as salivary contamination secondary to dribbling at the lip commissure, failure to achieve an airtight seal over the hair-bearing site, and the continuous movement of chin resulting in a loosening of dressing, contributed to this.

Another important consideration in the application of NPWT is the amount of pressure to be applied and whether in the continuous or intermittent modes. The median atmospheric pressure in our cohort was 125 mm of Hg. Moues et al. justified using this pressure because it increases blood flow by four times. Only eight studies have mentioned the dynamics of pressure adjustment [34]. All of these except one applied continuous pressure, while one study used continuous pressure for 24 h followed by intermittent pressure. The idea was to avoid excessive maceration to the surrounding normal skin. Reiter et al. used reduced pressure (75 mm of Hg) in two patients due to pain-related issues secondary to high pressure. [17]. Borgquist et al. measured the effect of negative pressure on microvascular blood flow to the wound and reported 80 mm of Hg (similar to 125 mm of Hg) to be an effective amount of pressure. Raising the pressure to above 125 mm of Hg has no added benefits rather in high-flow wounds where interstitial fluid or chyle output may be increased [35].

The wound healing response was variable in our cohort. None of these studies aimed at the complete closure of the wound. The intended use was to facilitate granulation tissue formation until subsequent reconstruction using skin graft or local flaps. Mir et al. labelled the response to be 100% when there was either complete closure or granulation tissue formation and otherwise a failed or 0 response [36]. The studies in cumulative series showed a mean response rate of 88.2% (82.2 ± 10.4). Only four studies documented a 100% response by NPWT, while six achieved a response of more than 80%. We had a different understanding here. Large wound defects may not necessarily be completely closed by NPWT when a patient has a background of an immunocompromised status. A response of more than 80% is still encouraging. Any large defect that is less than 80% healed needs additional steps that may be in the form of local or regional flaps to achieve the desired result within an adequate time span.

The nature and location of the wound have important roles in estimating wound healing. The mean healing time from our cohort was 14.4 days (range: 6–53 days). Only eight studies clearly mentioned the duration of wound healing. Pharyngocutaneous fistulas are usually noticed in post-laryngectomy patients with a history of previous irradiation and result in long healing periods. Diabetes mellitus, radiation-induced hypothyroidism, and malnourishment can further add up to the existing condition and thus result in even more delayed wound healing [37,38,39]. Dorneden et al. explored the use of NPWT in chylous fistula in three patients as a complication after neck dissection. Their results seemed promising, with a reduction in chylous output in two-to-eight days, but it still needs to be seen whether NPWT holds an effective role in managing high volume fistulas [14].

The timing of change of foam dressing is a debatable issue as well. The cumulative results from our selected studies showed a median foam change dressing time of 3.2 ± 1.39 days. The foam dressing has to be completely removed because it is a foreign body and granulation tissue ingrowth poses a problem. However, this timing may have to be adjusted depending on the rate of wound healing [13]. A dressing change at every third day obviates the need for multiple dressings on a daily basis. This has positive impact in terms of reduced financial burdens on hospitals, patients, and insurance companies.

Though the complications associated with NPWT are not very frequent and of low morbidity, serious incidents have been reported [5,40,41]. Among our cohort, six studies did not report any complication. Asher et al. reported incidents of bleeding and inadvertently retained sponges in the wound in three cases [11]. Umezawa et al. documented contact dermatitis, and Satteson et al. noticed infection, seroma, or hematoma in a small number of cases. One has to be careful and vigilant in the use of NPWT in chylous fistula cases because fluid volume losses may gradually disturb the electrolyte balance. The proximity of great vessels in the vicinity of chyle leakage poses a potential risk of life-threatening hemorrhage. There have been no reports of such hemorrhage regarding NPWT application in head and neck wounds [19,22]. Dorneden et al. suggested a method to minimize the risk by applying a white, non-porous polyvinyl alcohol foam to protect vessels from negative pressure effects [14].

Salvage surgery in head and neck cancer patients carries a greater risk of wound-related complications. More commonly, salvage laryngectomies have resulted in higher incidences (60–80%) of major complications such as pharyngocutaneous fistula or chyle leak [42]. A systematic review and meta-analysis by Shukla et al. showed a worse free flap survival rate (RR 1.48; *p* = 0.004) and complications (RR 1.84; *p* < 0.001) for previously irradiated patients [43]. Therefore, the management of closed versus fistulous wounds is a huge point of debate. Andrew et al. described closure of the fistulous tract as a difficult task secondary to radiation-induced fibrosis [9]. This conclusion was negated by Inatomi et al., who reported no significant difference (*p* = 0.07) in fistula closure for previously irradiated wounds. In particular, the presence of oro-cutaneous or pharyngocutaneous fistula did not make a difference (*p* = 0.93) in terms of the duration of wound healing, although the duration of application of NPWT was a bit prolonged in pharyngocutaneous fistulas. However, no comparisons between the conventional dressings and NPWT for fistula closure have been made [15].

This is the critical area where NPWT should be a part of surgeon’s armamentarium, particularly in a multidisciplinary set up where one could not afford wound-related complications resulting in prolonged hospital stays, additional costs, and delays in further treatment. Therefore, it is paramount to include NPWT therapy in the training program of residents and fellows, as well as to make a clear statement that NPWT is not only a “nurse” thing but a key pillar in the framework of multidisciplinary teams focusing on wound or better defect closure.

**Figure 4:**
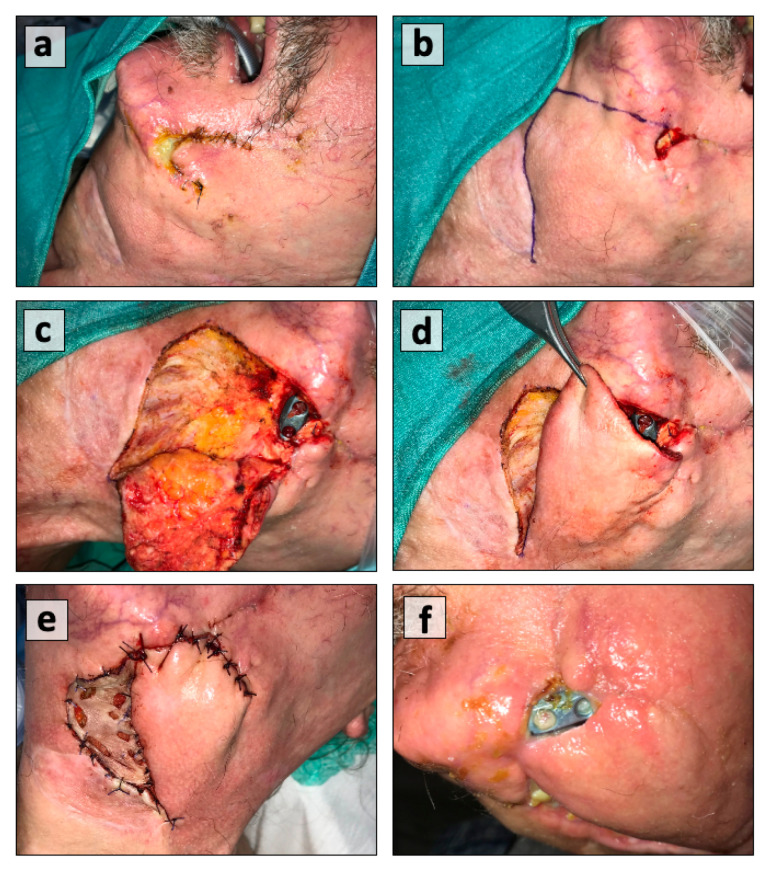
(**a**) Exposure of reconstruction plate post irradiation, (**b**) wound debridement and marking of advancement rotation flap, and (**c**) raising the flap; (**d**) adequacy of flap for plate coverage, (**e**) local infection resulting in flap dehiscence and plate exposure again, and (**f**) no wound coverage despite using NPWT.

NPWT has certain limitations that must be considered before its use. The anatomical variations of facial subunits and the dynamic nature of hair-bearing skin create a stressful situation of frequent dressing change due to loss of an airtight seal. Secondly, wounds incorporating part of the oral cavity usually expose the dressing to repetitive soakage secondary to salivary and food contamination. These factors tamper all efforts for wound management, resulting in unnecessary hospital stays and financial burdens. In our experience, patients’ non-compliance with the NPWT has also been observed. The continuous negative pressure sound from the device has impacted patients´ and their partners´ sleep patterns and routine activities, making it cumbersome for some individuals to tolerate the system. One has to consider all these factors to make NPWT a meaningful practice. Moreover, our experience has shown that NPWT cannot overcome chronic infections like an infected plate of the mandible. In particular, NPWT would certainly reduce the amount of putrid secretion and local infection, but chronic infection cannot be eradicated (Figure 4). The incorporation of vacuum-assisted therapy for complex and challenging wounds, particularly post chemoradiation, has dramatically changed the practice of wound care in the last 20 years.

The studies selected for this paper used NPWT as a method to treat complex head and neck wounds, but no detailed information about the duration and types of conventional methods used before the application of NPWT has been provided by most publications. This obviously limits the value of any outcome, and it must be considered a limitation in our attempt to gather as much information as possible from the published literature about NPWT in the head and neck. Similarly, sub-class analyses of types of fistula (oro-cutaneous vs. pharyngocutaneous) and the differences in their healing times and healing response have been missing. This further warrants future prospective studies comparing fistulous versus closed wounds in the background of other factors such as types of fistula, previous irradiation, and the impact of comorbidities on wound healing. Our study presented a comprehensive overview of NPWT, keeping all its merits and demerits in view, to guide physicians for its application.

## 4. Materials and Methods

### 4.1. Literature Search or Data Selection

We performed an extensive search through PubMed, Cochrane Library, Web of Science, EMBASE, Biomedical Literature Database (CBM), and Clinicaltrials.gov with reference to negative pressure wound therapy in head and neck tumors. Articles meeting the search criteria with keywords such as ‘negative pressure wound therapy’ or ‘NPWT’ or ‘vacuum-assisted closure’ or ‘VAT’ or ‘negative pressure dressings’ or ‘vacuum assisted closure’ or ‘VAC’ were included in the first instance, which concluded on 29.02.2020. The analysis included a systematic review, retrospective studies, literature reviews, case series, and case reports with 3 or more cases focusing on the management of head and neck wounds that exclusively involved the head, face, and neck zones published from 2000 to 2020.

### 4.2. Data Collection

Data on patients’ age, year of publication, gender, etiology, zone of involvement in the head and neck region, applied pressure by the device, wound healing response and duration, associated risk factors, dressing change protocols, wound sizes, duration of hospital stay, cost of the treatment, and associated complications were found. The selection of bias assessment was performed by 2 independent authors (P.D.B. and M.F.) using a tool developed by the National Heart, Lung, and Blood Institute at the National Institutes of Health. Different criteria were applied for the cohorts and case series to determine the quality of the studies. We found very few focused publications on the application of NPWT in the head and neck region following tumor surgeries. We had to extract data addressing cancer-related wounds in head and neck. The criteria for exclusion were the exclusive address of trauma and infection, cases such as necrotizing fasciitis or deep neck abscess, articles focusing on donor site wound management using NPWT, and case reports and case series reporting on less than 3 patients. The data extraction and quality assessment of all included studies were independently performed by 2 authors (M.F. and P.D.B.). Controversies were solved by discussion or consultation with other authors (R.S. and B.M.E.). All included papers were clinical studies focusing on the management of non-healing wounds of the head and neck after ablative tumor surgeries.

### 4.3. Data Analysis and Statistical Methods

The cohort was subjected to binary regression analysis to find any relationship between wound healing and associated risk factors or comorbidities. Wound healing was defined as granulation tissue formation after NPWT to cover more than 80% of the defect with no additional need for flap coverage, while less than 80% of wound coverage was labelled as incomplete healing that necessitated flap reconstruction. SPSS^®^ version 25 (IBM, Armonk, NY, USA) was used for statistical analysis. An independent t test was used to compare the means of 2 normally distributed groups. A *p*-value of < 0.05 was considered to be significant. The association between the groups was represented by scatter plot made by GraphPad Prism 8 (GraphPad Software, San Diego, CA, USA).

## 5. Conclusions

NPWT must be considered an excellent alternative to traditional dressings for the management of compromised head and neck wounds. The anatomical complexity of these areas along with risk factors such as previous radiation and DM has posed challenges to the treating physicians in terms of complete wound healing. It is of utmost importance to give prime focus to the management of DM as a modifiable factor by actively involving medics. The formation of granulation tissue beds, the obliteration of dead space, and the closure of fistulas are the primary outcomes of NPWT. Secondary outcomes should be decreased hospital stays, cost-effectiveness, and hospital burdens.

## Figures and Tables

**Table 1:** Demographics of the cohort, wound response, healing time, pressure dynamics, comorbidities, risk factors, and complications.

Author	Study Type	Year of Publication	N	Age (in Years)	M:F	Pressure Mode	Negative Pressure	Comorbidity	Previous Irradiation (N)	Healing Response	Complication
Andrew [9]	2006	Retrospective case series	12	67	8:4	-	125	DM *-2 CAD *-2 HTN *-7 Pulmonary disease-7	1	100	-
Andrew [10]	2008	Case series	3	67	2:1	-	150		2	66	
Asher [11]	2014	Retrospective cohort	108	64	87:28	Continuous	125	-	47	75	Bleeding Retained sponge
Asher [12]	2014	Retrospective cohort	12	63	6:7	Continuous	125	DM *-4 Hypothyroidism-6 Malnutrition-6	9	92	-
Dhir [13]	2009	Retrospective cohort	19	63	17:2	Continuous	110	DM *-8 HTN *-16 Malnutrition-9 CAD *-6 PVD *-7	10	84	-
Dorneden [14]	2019	Case series	3	66	1:2	Continuous	125	-	0	100	Electrolyte loss
Inatomi [15]	2019	Retrospective cohort	32	63	28:6	-	125	-	14	82	None
Lin [16]	2018	Retrospective series	31	52	29:2	-	100	DM *-5 HTN *-6	0	90	-
Reiter [17]	2013	Retrospective cohort	23	58	12:0	Continuous	125	HTN *-3	11	78	None
Rosenthal [18]	2005	Retrospective series	14	59	14:5	Continuous	125	-	0	86	None
Satteson [19]	2015	Retrospective cohort	69	66	45:25	Continuous	-	Smoking-30 HTN *-35 DM *-17	36	90	Infection Hematoma Seroma
Tian [20]	2016	Case series	4	55	3:1	-	-	-	0	-	None
Thierauf [21]	2018	Retrospective case series	20		8:5		70	DM *-3 PVD *-1 HIV *-1 RA *-1	7	110	None
Umezawa [22]	2018	Case series	17	67	10:1			-	6	100	Contact dermatitis
Yang [8]	2013	Case series	13	50	13:0	Continuous	125	DM *-4 HTN *-2 CVA *-1	0	92	None

* Abbreviations: N: number; M: male; F: female; diabetes mellitus; CAD: coronary artery disease; HTN: hypertension; RA: rheumatoid arthritis; HIV: human immunodeficiency syndrome; CVA: cerebrovascular accident; PVD: peripheral vascular disease.

**Table 2:** Univariate and multivariate binary regression analysis to assess wound healing response in the presence of risk factors.

Variable	Univariate Analysis			Multivariate Analysis		
	OR	*p*	95% CI	OR	*p*	95% CI
Sex (Female)	2.26	0.103	0.85–6.0	2.01	0.223	0.66–6.15
RT (Yes)	4.34	0.005	1.54–11.6	4.07	0.010	1.39–11.9
DM (Yes)	5.65	<0.001	2.14–14.9	5.62	0.001	2.01–15.6
RT and DM (Yes) *	10.6	<0.001	3.38–33.3			
Malnutrition (Yes)	1.34	0.671	0.35–5.21			
PVD (Yes)	1.17	0.889	0.13–10.2			
Hypothyroidism (Yes)	0.86	0.889	0.10–7.52			

* Abbreviations: RT: radiotherapy; DM: diabetes mellitus; PVD: peripheral vascular disease; OR: odds ratio; CI: confidence interval.

**Table 3:** Results of quality of assessment using National Institutes of Health Quality Assessment Tools [23].

Authors	Study Design	Clear Study Objective	Sample Size Justification	Association/Assessed Levels of Exposure on Outcome	Exposure Defined/Exposure Assessed Repeatedly	Clearly Define Outcome	Clearly Statistic Defined	Follow Up > 80%	Confounding Variables Measured	Rating
Andrew [9]	Retrospective case series	+/+	+	+/+/+	+/+	+	+	+	NR *	Good
Andrew [10]	Case series	+/+	NR *	+	+/+	CD *	+	+	NR *	Fair
Asher [11]	Retrospective cohort	+/+	+	+/+/+	+/+	+	+	+	+	Good
Asher [12]	Retrospective cohort	+/+	+	+/+/+	+/+	+	+	+	+	Good
Dhir [13]	Retrospective cohort	+/+	+	+/+/+	+/+	+	+	+	+	Good
Dorneden [14]	Case series	+	CD *	+/+/+	+/+	CD *	+	+	NR *	Fair
Inatomi [15]	Retrospective cohort	+/+	+	+/+/+	+/+	+	+	+	+	Good
Lin [16]	Retrospective series	+/+	+	+/+/+	+	+	+	+	CD *	Good
Reiter [17]	Retrospective cohort	+/+	+	+/+/+	+	+	+	+	+	Good
Rosenthal [18]	Retrospective series	+/+	+	+/+/+	+	+	+	+	+	Good
Satteson [19]	Retrospective cohort	+/+	+	+/+/+	+	+	+	+	+	Good
Tian [20]	Case series	+	CD *	+	+	+	+	+	NR *	Fair
Thierauf [21]	Retrospective case series	+	+	+	+	+	+	+	+	Good
Umezawa [22]	Case series	+	+	+/+	+	+	+	+	CD *	Good
Yang [8]	Case series	+	+	+/+/+	+	+	+	+	+	Good

* Abbreviations: CD: cannot determine; +: criterion met; NR: not recorded.

## Data Availability

Data is contained within the article and is available on request.
